# Supplementary material for: NK2R signaling governs intestinal lipid mobilization and mucosal inflammation
Source: bioRxiv. 2025 Nov 21:2025.11.21.689675. Preprint. [Version 1] doi: 10.1101/2025.11.21.689675 (PMC12667774; doi:10.1101/2025.11.21.689675)
Supplement: 1 [file NIHPP2025.11.21.689675V1-supplement-1.pdf]

bioRxiv preprint doi: https://doi.org/10.1101/2025.11.21.689675; this version posted November 23, 2025. The copyright holder for this preprint (which was not certified by peer review) is the author/funder, who has granted bioRxiv a license to display the preprint in perpetuity. It is made available under aCC-BY 4.0 International license.

Supplementary Table 1: qPCR primers used in this study.

qPCR primers used in this study	Source	Sequence
qPCR Forward primer for *Tacr1*	IDT	TCATCGTGGTGACTTCCGTG
qPCR Reverse primer for *Tacr1*	IDT	TGTGGACTGCGTAGGTGAAG
qPCR Forward primer for *Tacr2*	IDT	AGGTTTCGCTCTGGATTCCG
qPCR Reverse primer for *Tacr2*	IDT	TTGGTAGCCTCAGAGTGGGT
qPCR Forward primer for *Tacr3*	IDT	AGCATTTCGCTGGTGTCCTT
qPCR Reverse primer for *Tacr3*	IDT	TGTGGGATGAAACCTGGTGG
qPCR Forward primer for *Adipoq*	IDT	TGTTCCTCTTAATCCTGCCCA
qPCR Reverse primer for *Adipoq*	IDT	CCAACCTGCACAAGTTCCCTT
qPCR Forward primer for *Pparg*	IDT	CCAGCTCTACAACAGGCCTCAT
qPCR Reverse primer for *Pparg*	IDT	GTGGAAGCCTGATGCTTTATCC
qPCR Forward primer for *Lep*	IDT	AAGCAGTGCCTATCCAGAAAGTCC
qPCR Reverse primer for *Lep*	IDT	ATAGACTGCCAGAGTCTGGTCCAT
qPCR Forward primer for *Actb*	IDT	CATCCTCTTCCTCCCTGGAGAAGA
qPCR Reverse primer for *Actb*	IDT	ACAGGATTCCATACCCAAGAAGGAAGG
qPCR Forward primer for *Rplp0*	IDT	AGATTCGGGATATGCTGTTGGC
qPCR Reverse primer for *Rplp0*	IDT	TCGGGTCCTAGACCAGTGTTC
qPCR Forward primer for *Hprt*	IDT	CTGGTGAAAAGGACCTCTCGAAG
qPCR Reverse primer for *Hprt*	IDT	CCAGTTTCACTAATGACACAAACG
qPCR Forward primer for *Vil1*	IDT	AGTCATCACTCCTCGGCTCT
qPCR Reverse primer for *Vil1*	IDT	GAAAACGTCCACTTTCGGGC

Supplementary Table 2: Genotyping primers used in this study.

Genotyping primers used in this study	Source	Sequence
Forward primer for genetyping *Tacr2-/-*	IDT	GGTTTGATCTATGTCTGAGAAATGG
Reverse primer for genotyping *Tacr2-/-*	IDT	CCTTTCTGTTTGCTCTCAAGAAG
Forward primer for genetyping *Tacr2*fl/fl	IDT	TTGGATCATGGTGTGGCTGT
Reverse primer for genotyping *Tacr2*fl/fl	IDT	CTCAAGAAGTGGACAGAGGCA
